# Targeting C3a and C5a Signaling—A Game Changer for Cancer Therapy?

**DOI:** 10.3390/biology14111491

**Published:** 2025-10-25

**Authors:** Hunter Hudgins, Valeria Molina, Stanley Wiernicki, Kenneth Okwuegbe, Xiaodong Feng, Hongbin Wang

**Affiliations:** 1 Master Program of Pharmaceutical Sciences College of Graduate Studies, California Northstate University, 9700 West Taron Dr., Elk Grove, CA 95757, USA; hunter.hudgins10766@cnsu.edu (H.H.); valeria.mxc267@gmail.com (V.M.); stanley.wiernicki11365@cnsu.edu (S.W.); 2 PhD Program of Pharmaceutical Sciences College of Graduate Studies, California Northstate University, 9700 West Taron Dr., Elk Grove, CA 95757, USA; 16260@cnsu.edu; 3 Department of Pharmaceutical and Biomedical Sciences College of Pharmacy, California Northstate University, 9700 West Taron Dr., Elk Grove, CA 95757, USA; xfeng@cnsu.edu

**Keywords:** cancer, complement, C3a/C3aR signaling, C5a/C5aR signaling, immunotherapy, tumor microenvironment (TME)

## Abstract

**Simple Summary:**

Cancer is one of the deadliest diseases worldwide. When cancer cells spread to other parts of the body, the disease is hard to treat. One of our body’s defense systems, called the complement system, usually fights off infections, but recent studies show that it can also help cancer cells grow and spread. We examined research on two proteins from this system, C3a and C5a, and found that they can make cancer worse. They do this in two ways: directly helping cancer cells grow and multiply, and weakening the immune system’s ability to fight them. This means that C3a and C5a can help cancer cells thrive and spread to other parts of the body. This discovery opens up new possibilities for cancer treatment. One potential solution is to use medicines that inhibit those two proteins, which could help treat cancer more effectively. In conclusion, the complement system’s components, C3a and C5a, play a significant role in promoting cancer cell growth and spread. By understanding their mechanisms, we can explore new approaches to cancer treatment, such as using inhibitors to block their effects. This promising avenue of research holds potential for improving cancer therapy and patient outcomes.

**Abstract:**

Emerging evidence reveals a significant shift in understanding the complement system’s role in cancer, where activation of a complement within the tumor microenvironment (TME) fuels tumor growth and metastasis instead of suppressing it. Research highlights C3a and C5a anaphylatoxins as key drivers of cancer progression, showing that the blockade of their signaling pathways can inhibit tumor growth and metastasis. By interacting with immune cells in the TME, including tumor-associated macrophages (TAMs), T cells, and myeloid-derived suppressor cells, C3a and C5a promote immunosuppression, thereby driving cancer cell proliferation, angiogenesis, and metastasis. However, conflicting findings persist, despite growing evidence supporting the role of C3a and C5a in tumor progression and the potential therapeutic benefits of targeting pathological complement activation. This paper presents a systematic review of studies examining the activation of the complement system and the role of the C3a and C5a signaling pathways in the TME, focusing on their effects on tumor progression, their interactions with TME components, and the potential for targeting these signaling pathways to boost anti-tumor immune responses.

## 1. Introduction

The tumor microenvironment (TME) is a complex, dynamic ecosystem consisting of diverse cellular and non-cellular components that interact with neoplastic cells, which might promote tumor progression and metastasis while evading immune and therapeutic responses. This intricate network comprises immune cells, endothelial cells, cancer-associated fibroblasts, pericytes, stromal cells, blood vessels, extracellular matrix (ECM) proteins, signaling molecules, and metabolic factors, which engage in bidirectional interactions influencing tumor growth, invasion, angiogenesis, and immune evasion. Cancer cells can exploit regulatory receptors and molecules, creating a favorable environment for their survival and metastasis. Understanding the interplay between cancer cells and the TME is crucial for elucidating cancer progression mechanisms and developing novel therapeutic strategies [1,2,3,4].

The complement system, a crucial component of the innate immune system, plays a pivotal role in modulating adaptive immunity, maintaining tissue homeostasis, synaptic pruning, neuroprotection, and other physiological functions [5]. In response to pathogen invasion, the activation of the complement system facilitates the phagocytosis of pathogens by opsonization and lysis of pathogens through the generation of membrane attack complex (MAC). The tumor microenvironment is heavily regulated by the complement system. Complement activation in response to tumors can paradoxically create an environment that supports tumor progression by promoting an immunosuppressive microenvironment, inducing angiogenesis, and activating cancer-related signaling pathways [6,7]. Recent studies have highlighted the complement system’s role in promoting tumor progression and metastasis within the TME through immunosuppressive effects, rather than inhibiting tumor progression. Groundbreaking research by Markiewski et al. demonstrated that complement activation plays a crucial role in shaping the TME by inhibiting T cell functions, evidenced by the fact that C3 deficiency or C5aR inhibition impaired tumor growth and enhanced the infiltration of CD8^+^ T cells into the TME. These findings highlight the importance of complement activation in the TME and its potential as a therapeutic target [8]. Additional studies have shown that complement activation components can directly drive tumorigenesis. For example, C5a generated from intracellular complement C5 by Cathepsin D can interact with intracellular C5aR1 to form a complex with KCTD5/cullin3/Roc-1 and β-catenin to promote β-catenin stabilization, which is associated with poor prognosis for cancer. High levels of C5aR1, C5a, and Cathepsin D are closely correlated with elevated β-catenin levels and poor clinical outcomes [9,10]. A mounting body of evidence supports the notion that complement activation in TME significantly contributes to the advancement of tumor progression, prompting us to propose that targeting anaphylatoxin C3a and C5a signaling pathways may improve cancer immunotherapy.

However, research on anaphylatoxins C3a and C5a has yielded mixed results. Some studies suggest that these molecules promote tumor progression, supporting the premise that pathological complement activation could be a therapeutic target. Other research indicates that anaphylatoxins can inhibit tumor growth by boosting immune functions, highlighting the complexity of this process [11,12,13,14]. This review will discuss the role of complement activation in the TME, focusing on C3a and C5a’s involvement in cancer progression. It will explore how these molecules drive tumor growth, and suggests that targeting C3a and C5a alongside standard therapies like chemotherapy, radiotherapy, and immune-checkpoint inhibitors could enhance cancer treatment outcomes [15,16].

## 2. Generation of Anaphylatoxins C3a and C5a in the TME

The complement system is a crucial effector arm of innate immunity, comprising over 50 membrane-bound and soluble proteins [17]. It activates through three conventional pathways: the classical pathway, which is antibody-dependent and initiated by antigen-bound IgG or IgM interacting with C1q; the lectin pathway, which recognizes pathogen-associated molecular patterns via mannose-binding lectin (MBL) and other pattern recognition receptors; and the alternative pathway, involving spontaneous C3 hydrolysis and stabilization by properdin. All three canonical complement cascades converge to form C3 convertases, cleaving C3 into C3a and C3b and further cleaving C5 by C5 convertases into C5a and C5b, ultimately leading to membrane attack complex (MAC) C5b-9 formation and pathogen lysis [18].

Research has shown that all three canonical complement pathways (classical, lectin, and alternative) contribute to complement activation, resulting in the production of anaphylatoxins C3a and C5a in TME [8,19,20]. Notably, multiple non-canonical mechanisms have been identified to trigger complement activation [21,22,23,24,25]. Anaphylatoxins C3a and C5a can be generated by soluble and membrane-bound proteases, including those found in coagulation, fibrinolysis, and phagocytic cells [25,26]. Notably, trypsin can cleave C5 into biologically active C5a-like fragments [24], and C5 can also be cleaved and activated by various proteases, including plasmin, cathepsins, and thrombin [9,26,27,28,29,30].

**Figure 1:**
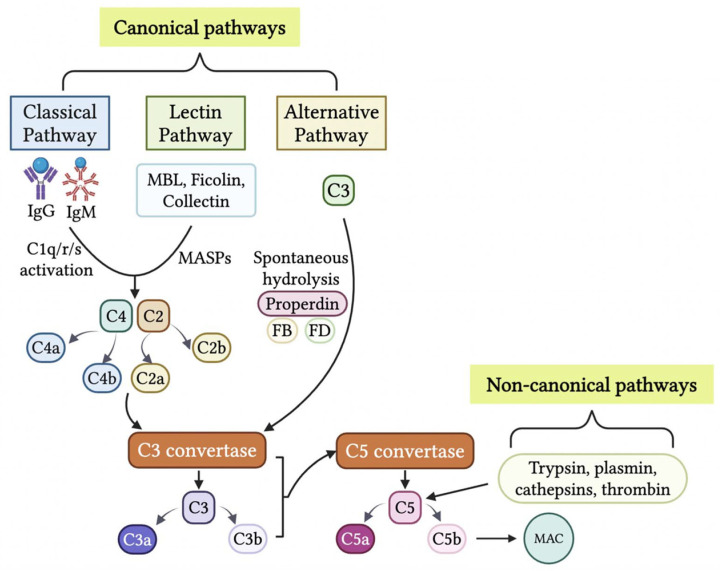
**Generation of anaphylatoxins C3a and C5 in the tumor microenvironment (TME).** The production of C3a and C5a are initiated through cascades from the canonical pathways or non-canonical pathways. The three canonical pathways, classical pathway, lectin pathway, and alternative pathway, through differing activation cascades, result in the production of C3 convertases. C3 convertases cleave C3 into C3a and C3b. C3b combines with C3 convertase to create C5 convertase. C5 convertase cleaves C5 into C5a and C5b. C5b results in initiation of the MAC. The non-canonical pathways consist of C3 and C5 being cleaved by proteases such as thrombin, cathepsins, plasmin, and trypsin. This results in the production of anaphylatoxins C3a and C5a within the TME. The figure was created with BioRender.com.

C5 can be cleaved in a C3-independent manner by plasmin activated by urokinase (uPA)-expressing macrophages, promoting mouse squamous carcinogenesis [28]. Consistent with this finding, C5-deficient, but not C3-deficient, mice show markedly reduced colorectal tumorigenesis, highlighting the importance of C3-independent C5 activation. Thrombin produced in tumors also exhibits potent pro-tumor activity and supports early, inflammation-driven tumorigenesis in colorectal cancer [31]. These findings suggest that thrombin may serve as another protease capable of cleaving and activating C5 within the tumor microenvironment [29,32]. In summary, the generation of anaphylatoxins C3a and C5a could be due to the activation of canonical or noncanonical mechanisms in the TME (Table 1 and Figure 1).

## 3. Anaphylatoxins Directly Drive Tumor Cell Proliferation and Progression

Research has shown that anaphylatoxin signaling can directly drive cancer progression and metastasis. A study by Chen et al. found elevated C5aR expression in breast cancer tissues. Mice lacking or treated by blocking C5aR showed reduced tumor growth, linked to changes in the MAPK/p38 signaling pathway and increased p21 expression. The study suggests C5a/C5aR signaling promotes tumor growth by suppressing anti-tumor immunity and enhancing cancer cell growth. However, the complexity of the tumor microenvironment in C5aR ablation mouse models with xenografted breast cancer cells may limit their use in studying direct C5a/C5aR effects on tumor progression [34]. Lu et al. studied the role of complement proteins C3a and C5a in breast cancer proliferation. They found that C5a, but not C3a, enhanced breast cancer cell growth by activating RGC-32. This effect was blocked by inhibiting C5aR or silencing RGC-32. Furthermore, the study showed that C5a promotes proliferation through the Akt1-RGC-32 pathway, as evidenced by suppression of RGC-32 expression with the Akt inhibitor Ly294002 [35]. In a separate study, Xiong et al. examined the role of C3a and C5a in multiple-myeloma cell migration, invasion, and adhesion. They found that patients with multiple myeloma had higher plasma levels of C3a, C5a, and their receptors compared to healthy donors. In vitro experiments showed that C3a and C5a activated the MEK/ERK pathway and increased nuclear translocation of Nrf2 in multiple-myeloma cell lines [36].

Ding et al. discovered a novel intracellular mechanism driving colorectal cancer progression. They found that cathepsin D cleaves complement C5 to generate C5a, which interacts with C5aR1, stabilizing β-catenin via a protein complex. Elevated β-catenin levels are associated with poor outcomes in colorectal cancer. The study also revealed correlations between C5aR1, C5a, cathepsin D, and β-catenin levels, predicting poor prognosis. These findings suggest that targeting the C5a/C5aR1 pathway may be a promising therapeutic approach for colorectal cancer [9,10].

Liu et al. investigated the role of C5aR1 in anaplastic thyroid carcinoma (ATC) progression. They found that C5aR1 levels were elevated in ATC tumor samples and correlated with poorer survival outcomes. C5aR1 knockdown reduced ATC cell proliferation, migration, and invasion, while C5aR1 overexpression had the opposite effect. The study also showed that C5aR1 regulated proinflammatory pathways, including TLR1, TLR2, and MyD88. In mouse models, silencing C5aR1 suppressed tumor growth and lung metastasis. Additionally, miR-335-5p was found to negatively regulate C5aR1. The findings suggest that the miR-335-5p/C5aR1/TLR1/2 axis may be a potential therapeutic target for ATC [37].

**Figure 2:**
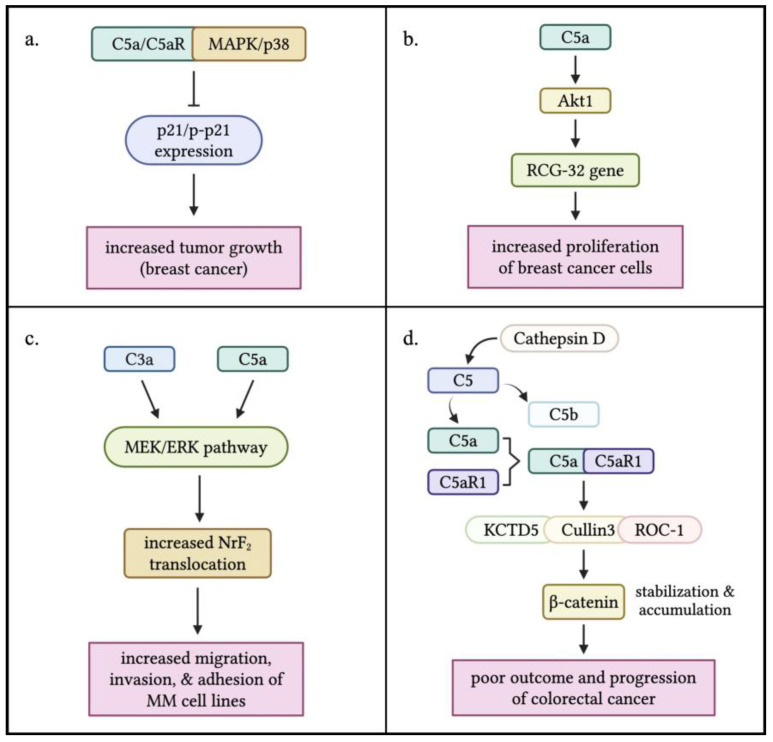
**Direct effects of anaphylatoxins C3a and C5a on tumor cell progression.** (**a**) Interaction between C5a/C5aR signaling and the MAPK/p38 pathway results in the inhibition of p21/p-p21 expression, which was found to enhance tumor growth in breast cancer. (**b**) C5a stimulation triggers the activation of the RGC-32 gene by Akt1 leading to an increased proliferation of breast cancer cells. (**c**) Both C3a and C5a activate the MEK/ERK pathway, resulting in an increased nuclear translocation of NrF2 that induces the increased migration, invasion, and adhesion of multiple myeloma (MM) cells. (**d**) C5a is produced within the TME through the cleavage of C5 by Cathepsin D, a non-canonical pathway. Interaction between C5a and C5aR1 triggered the formation of a complex with KCTD5, Cullin3, and ROC-1, which stabilized and accumulated -catenin in tumor cells. This leads to a poor outcome, and progression of colorectal cancer. The figure was created with BioRender.com.

Although studies have demonstrated the direct effects of anaphylatoxin on tumor progression, many other studies found no evidence of the direct effects of C3a or C5a on tumor progression [8,19,23,38]. The disparate findings regarding anaphylatoxin’s direct effects on tumor progression may stem from heterogeneity in receptor expression profiles across distinct tumor cell lineages. C5aR is typically not expressed on epithelial cells, but can be induced in response to inflammation and certain cancers [39,40]. Research by Nitta et al. found that, in cancerous settings, C5aR is aberrantly expressed on various epithelial tissues, including the esophagus, stomach, colon, liver, pancreas, bladder, bile duct, prostate, and mammary glands [39,40,41,42,43,44]. This suggests that C5aR expression in these tissues may be a result of malignant transformation, highlighting a potential link between C5aR and cancer progression [41], which may drive tumor growth and metastasis in the TME. A comprehensive summary of the relevant studies can be found in Table 2 and Figure 2.

## 4. Anaphylatoxin Signaling Pathways Contribute to Tumor Progression by Regulating Immune Responses in the TME

C3a and C5a signaling pathways have been recognized as one of the key players in fostering rather than defending against cancer progression and metastasis in the TME. They mediate chronic inflammation by suppressing the immune effector cells in the TME responsible for immunosurveillance [6,45]. M2 tumor-associated macrophages express high levels of C3aR and C5aR, binding to their respective anaphylatoxins, C3a and C5a, to promote immunosuppression, angiogenesis, and activation of signaling pathways associated with cancer progression [6,46,47].

### 4.1. The C3a/C3aR Axis Contributes to Cancer Progression by Regulating Immune Responses in TME

Macrophages play a dual role in the TME, either contributing to anti-tumor immunity as M1 subtype or promoting tumor growth as TAMs of the M2 subtype. M1 macrophages possess anti-tumor properties and can enhance anti-tumor immunity and inhibit tumor progression. However, within the immunosuppressive TME, the polarization of macrophages towards an M1-like phenotype is often subverted. Factors such as hypoxia, tumor-derived cytokines (e.g., IL-4 and IL-13), and metabolic alterations contribute to the polarization of macrophages towards an M2-like phenotype [48]. The anti-inflammatory M2 phenotype is indirectly tumor-promoting. In many solid tumors, macrophages are skewed towards an M2-like phenotype, characterized by the expression of markers such as CD206 and CD163. These M2-like macrophages promote tumor growth and metastasis through various mechanisms, including the secretion of immunosuppressive cytokines (e.g., IL-10 and TGFβ), upregulation of immune checkpoints, promotion of angiogenesis, and remodeling of the extracellular matrix to facilitate tumor invasion [49,50,51]. More importantly, M2-like macrophages contribute to immune evasion by inhibiting T cell function and promoting the recruitment of regulatory T cells [52]. C3a/C3aR signaling has been linked to recruitment of macrophages to the TME to suppress immune reaction and cause tumor progression, as presented by the following experimental examples.

Ah-Pine et al. investigated complement activation and the upregulation of C3a and its receptor C3aR in glioblastoma (GBM). Glioblastoma tissue analysis revealed high levels of complement fragment C3a within the parenchyma, suggesting alternative pathway activation via a Bb-dependent mechanism. Immunohistochemical analysis showed that C3aR-expressing cells were predominantly located around blood vessels and co-expressed macrophage markers CD68 and CD45. Notably, these C3aR-positive cells were distinct from the GBM/A4 cell population. Further characterization revealed that C3aR was frequently co-expressed with α2 integrin (CD18) and CD68, confirming the macrophage identity of these cells. The study also identified C3aR-positive TAMs that expressed CD163, an M2 phenotype marker. These C3aR TAMs strongly expressed vascular endothelial growth factor (VEGF), potentially driving robust angiogenesis and metastasis observed in various cancer models. In vitro experiments using PMA-differentiated THP1 cell lines showed that transforming growth factor-beta 1 (TGF-β1) is a potent growth factor that upregulates VEGF, C3, and C3aR in TAMs [20]. These findings highlight the crucial role of C3a in recruiting and differentiating monocytes into anti-inflammatory macrophages (M2). This suggests that C3aR expression by TAMs in glioblastoma may contribute to immunosuppression and promote tumor growth, consistent with observations in melanoma and sarcoma mouse models [53,54]. This study provides new insights into the pathogenesis of glioma and opens potential therapeutic avenues, such as targeting C3aR with antagonists, for glioblastoma.

Davidson et al. used single-cell RNA sequencing to study the dynamic stromal niche that fosters tumor growth. By examining the stromal compartment in mouse melanoma and lymph nodes at various stages of tumor development, they uncovered a complex interplay between cells within the evolving tumor. The analysis revealed three distinct stromal populations in the TME, S1 (immune), S2 (desmoplastic), and S3 (contractile), each with unique functional signatures that were conserved across mouse and human tumors. Notably, the immune population (S1) specifically expressed a high level of complement component C3. This expression was specific to S1 cells, even in the broader tumor context and across multiple tumor types. The C3 cleavage product C3a was found to regulate immune populations by recruiting C3aR^+^ macrophages. Disrupting the C3a/C3aR interaction between S1 cells and myeloid cells by neutralizing C3a in established tumors significantly slowed tumor growth. This effect was accompanied by reduced macrophage infiltration and increased Ly6C^+^ monocyte subpopulations. Furthermore, anti-C3a treatment led to a higher frequency of CD8^+^ tumor-infiltrating T cells, suggesting that suppressive myeloid cell recruitment contributes to CD8^+^ T cell suppression. Similar findings were observed in human melanoma and head and neck cancers, indicating that the C3a/C3aR axis mediating cancer-associated fibroblast (CAF)–macrophage crosstalk is conserved across multiple tumors and species [54].

Magrini et al. found that the lectin pathway and C3a receptor (C3aR) contribute to tumor promotion in sarcomagenesis. Deficiencies in C3, C4, and MBL1/2 delayed tumor appearance, while C3aR deficiency had a similar effect. In contrast, C5aR1 and C5aR2 deficiencies had no impact. The study suggests that C3a/C3aR signaling is the primary driver of complement-mediated sarcoma promotion. These findings highlight the importance of C3a/C3aR in sarcomagenesis and metastasis. Moreover, the study found that C3aR is mainly expressed on tumor-infiltrating immune cells, such as macrophages and monocytes. C3aR deficiency reduced macrophage recruitment, shifted macrophage polarization towards anti-tumor phenotypes, and boosted T cell-mediated anti-tumor responses. In human sarcoma samples, C3aR expression correlated with poor disease-free and metastasis-free survival. Notably, combining C3 or C3aR deficiency with anti-PD-1 treatment improved therapeutic efficacy, reducing tumor growth and metastasis. Pharmacological inhibition of C3aR also showed promising results when combined with anti-PD-1 treatment, highlighting the potential benefits of targeting C3aR in sarcoma therapy [19].

Gong et al. found that C3a promotes medulloblastoma progression by activating astrocytes through TNF-α. Elevated C3 and C3a levels were detected in medulloblastoma tissues. C3a enhanced astrocyte-mediated tumor growth by increasing GFAP expression, which was inhibited by the C3aR antagonist SB290157. The C3a/C3aR pathway activates the p38 MAPK pathway, leading to TNF-α production and tumor growth. Targeting the C3a-TNF-α axis may be a potential therapeutic strategy. Additionally, high TNF-αR expression correlated with poorer overall survival rates in 632 patient cases, suggesting that TNF-αR signaling promotes medulloblastoma progression [38].

Research by Shu et al. revealed that C3a/C3aR signaling promotes tumor cell metastasis by activating carcinoma-associated fibroblasts (CAFs). Binding C3a to C3aR triggers the PI3K/AKT signaling pathway, driving CAF activation. In human invasive breast cancer, higher C3 expression is associated with increased CAF activation markers and effectors. Disrupting C3aR signaling, either through genetic deletion or pharmacological inhibition, significantly reduces breast cancer metastasis to the lung. These findings suggest the C3a/C3aR axis as a potential therapeutic target for limiting breast cancer spread [55].

Wang et al. found that complement signaling inhibits anti-tumor immunity in melanoma. C3-deficient mice showed slower tumor growth, and the enhanced anti-tumor response was mediated by CD8^+^ cytotoxic T lymphocytes (CTLs). Complement signaling suppressed IL-10 production in CD8^+^ T cells, which is crucial for T cell proliferation and cytotoxic activity. Blocking C3a and C5a signaling restored IL-10 production and suppressed tumor growth. Inhibiting C3aR and C5aR using antagonists slowed tumor growth, and combining them with anti-PD-1 therapy enhanced the anti-tumor response. These findings suggest that C3aR and C5aR on CD8^+^ T cells may function as immune-checkpoint-like receptors, presenting potential targets for cancer immunotherapy [7,56].

Nabizadeh et al. found that C3aR promotes melanoma progression in a murine model. Mice lacking C3aR showed slower tumor growth, smaller tumor sizes, and a 70% increase in survival rate. C3aR antagonism also inhibited established melanoma growth. The absence of C3aR increased tumor-infiltrating neutrophils and CD4^+^ T lymphocytes, particularly the Th1, Th2, and Th17 subsets, while reducing macrophages. Neutrophils played a crucial role in the anti-tumor effects. The study suggests that C3aR is a potential therapeutic target for multiple cancers, including melanoma, colon cancer, and mammary carcinoma [53].

In a complementary study, Zha et al. showed that tumor-cell-derived C3 can actively suppress anti-tumor immunity by driving the accumulation and immunosuppressive activity of tumor-associated macrophages. This effect results from the intracellular activation of C3, which limits CD8^+^ T cell infiltration and function through a C3aR-dependent, but C5aR-independent, mechanism. Notably, deleting C3 expression in tumor cells enhanced the efficacy of anti-PD-L1 treatment, suggesting that tumor-cell-derived C3 may be a promising therapeutic target for cancer immunotherapy [57].

**Figure 3:**
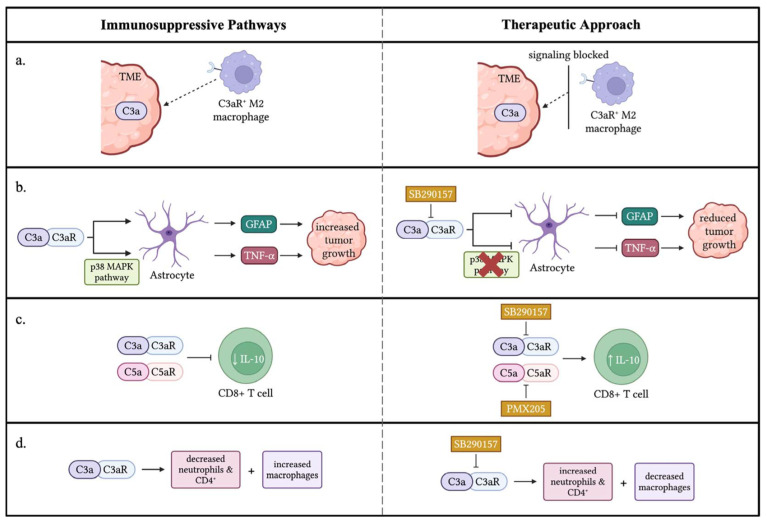
**Immune-suppressive effects of C3a/C3aR signaling in the TME.** (**a**) C3a in the TME attracts M2 macrophages that express the C3aR receptor promoting tumor progression. A therapeutic approach would block the signal between C3a and C3aR to decrease the migration of M2 macrophages to the TME. (**b**) Two pathways were displayed through which C3a/C3aR signaling enhances the ability of astrocytes to promote tumor growth through increased GFAP and TNF-α. A potential treatment, such as SB290157, a C3aR antagonist, could be utilized to block C3a/C3aR signaling inhibiting GFAP and TNF-α expression from astrocytes. (**c**) C3a/C3aR and C5a/C5aR signaling inhibit IL-10 production by CD8^+^ T cells. SB290157 and PMX205 inhibited anaphylatoxins’ signaling from blocking the IL-10 production of CD8^+^ T cells, resulting in a potential therapeutic approach. (**d**) C3a/C3aR signaling results in decreased neutrophils and CD4^+^ T cells within the TME and an increased number of macrophages, resulting in tumor growth. A potential treatment could be used to inhibit C3a/C3aR signaling, thus increasing the number of neutrophils and CD4^+^ T cells within the TME while decreasing macrophages to diminish tumor growth. ↓: decrease; ↑: increase. The figure was created with BioRender.com.

A study by Jackson et al. revealed that complement C3 plays a pivotal role in the development and growth of cutaneous squamous cell carcinoma (cSCC). Importantly, C3 activation fragments were detected within both human and murine tumors, where they were closely associated with infiltrating macrophages. In the DMBA-TPA model of cutaneous squamous cell carcinoma (cSCC), which depends on chronic inflammation, loss of C3 provided protection against tumor development. This protective effect, however, was not observed when tumors arose in the absence of inflammatory triggers. Interestingly, deficiency of C3aR1, C5aR1, or C5aR2 produced effects opposite to those observed with C3 deficiency, underscoring the complexity of complement receptor signaling in cancer. The findings suggest that C3 activation products, such as iC3b, C3b, and C3d, rather than anaphylatoxins C3a and C5a, can promote tumor growth and epithelial hyperplasia by interacting with the receptor CR3 on infiltrating myeloid cells, suggesting that complement C3 drives inflammatory skin carcinogenesis independently of complement C5 [58]. The immunologic regulation of the C3a/C3aR signaling pathway in TME is summarized in Table 3 and Figure 3.

### 4.2. The C5a/C5aR Axis Contributes to Cancer Progression by Modulating Immune Cell Functions in TME

Similarly to the C3a/C3aR signaling pathway, the C5a/C5aR signaling pathway also has been revealed to inhibit immune functions in TME, causing tumor progression. Markiewski et al. discovered that the activation of the complement system promotes tumor growth rather than inhibiting it. They found that C5a in the tumor microenvironment enhances tumor growth by suppressing CD8^+^ T cell-mediated anti-tumor responses. Inhibiting C5aR signaling boosted CD8^+^ T cell activity and reduced tumor growth. This was associated with decreased recruitment of myeloid-derived suppressor cells (MDSCs) into tumors. Using a TC-1 cervical cancer model, they showed that C3 cleavage products were deposited along tumor vasculature, indicating local complement activation. Tumor growth was reduced in C3- and C4-deficient mice, but not in factor B-deficient mice, suggesting classical or lectin pathway activation contributes to C3 activation. C1q deposition patterns suggested the classical pathway’s involvement. Blocking C5aR signaling impaired tumor growth, and C5aR-deficient mice had smaller tumors. Mechanistic studies revealed that C5a acts on host cells, not tumor cells, and that CD8^+^ T cells play a key role in controlling tumor growth. Tumors with blocked C5aR signaling increased CD8^+^ T cell infiltration, and depleting CD^8+^ T cells in C5aR-deficient mice increased tumor growth. Further research revealed that C5a plays a role in the migration of polymorphonuclear myeloid-derived suppressor cells (PMN-MDSCs) into tumors. MDSCs from C5aR-deficient mice showed impaired ability to suppress T cell proliferation compared to those from control mice. This suggests that C5a not only facilitates MDSC migration into tumors, but also enhances their immunosuppressive function. Additionally, C5a increases the production of reactive oxygen species (ROS) and reactive nitrogen species (RNS) by monocytic MDSCs (MO-MDSCs), further contributing to immune suppression [8].

Corrales et al. found that lung cancer cell lines produce higher levels of C5a than non-malignant lung epithelial cells. They also detected increased C5a levels in plasma from non-small-cell lung cancer (NSCLC) patients. Using a murine syngeneic lung cancer model, they showed that C5a contributes to tumor growth by creating a pro-tumor microenvironment. Blocking C5aR significantly reduced tumor growth, while C5a did not directly affect cancer cell proliferation in vitro. Further investigation revealed that C5a promotes angiogenesis, as evidenced by increased tube-like structure formation in HUVECs and enhanced micro-vessel formation in Matrigel plugs. Blocking C5aR reduced myeloid-derived suppressor cells (MDSCs) in the spleen of 3LL tumor bearing mice and decreased expression of immunosuppressive genes, including ARG1, CTLA-4, IL-6, IL-10, LAG3, and PD-L1 within tumors. These findings suggest that C5a creates an immunosuppressive tumor microenvironment, promoting tumor growth [23], supporting the rationale of blockade of C5aR to increase the efficacy of future therapeutic strategies for cancers.

Nunez-Cruz et al. developed transgenic mice to study the role of C3 and C5aR in epithelial ovarian cancer (EOC). They found that C3 deficiency impaired ovarian tumor development and growth. Analyzing tumor-infiltrating leukocytes, they observed no differences in overall leukocyte composition, but noted increased CD^8+^ T cells and decreased FoxP^3+^ CD^4+^ T cells in C3-deficient mice. C3 deficiency also leads to dampened cellular effector mechanisms, with reduced cytokine production (IL-12, IL-10, and IFN-γ) from macrophages, T cells, and B cells. Additionally, tumor microvascular density decreased in C3-deficient mice. C5aR inhibition impairs tube formation through the VEGF165 mediated pathway. Interestingly, C5aR knock-out models show compromised carcinogenesis and neo-angiogenesis without impacting immune cell infiltration. Combination knockouts of C3 and C5aR also diminished tumor growth, vascularization, and VEGF activity. This study reveals a novel mechanism of VEGF regulation through inhibition of the anaphylatoxin C5a. In addition, the data suggested that the classical or lectin complement activation pathway is involved in progression of epithelial ovarian cancer model [33].

Vadrevu et al. used a mouse model of breast cancer to show that C5a receptor 1 (C5aR1) promotes lung and liver metastasis by suppressing T cell responses in the lungs. This suppression is mediated by C5aR’s ability to recruit myeloid-derived suppressor cells (MDSCs) and generate regulatory T cells (T_regs_), as well as regulate the production of immunosuppressive cytokines like TGFβ and IL10. Blocking C5aR increased the recruitment of CD^4+^ and CD^8+^ T cells and induced Th_1_/T_c1_-biased T cell responses. The study found that C5aR inhibition or genetic ablation decreased MDSC infiltration in premetastatic niches, such as the lungs and liver, and reduced the number of CD11b^+^ cells producing TGFβ and IL10. Additionally, C5aR blockade increased the number of IFNγ-expressing CD4^+^ T cells and reduced IL4-producing CD^4+^ T cells, resulting in a higher Th_1_/Th_2_ ratio. Notably, the study’s relevance to human breast cancer was supported by the identification of MDSCs and complement deposition in tumor-draining lymph nodes of breast cancer patients. The findings suggest that targeting C5aR could be a potential therapeutic strategy to enhance anti-tumor immunity in breast cancer [59].

Medler et al. investigated the role of C5a in squamous carcinogenesis using the K14-HPV16 transgenic mouse model. The researchers found that C5aR1^+^ leukocyte infiltration occurred during neoplastic progression. C5a deposition was identified as an early and prominent feature of benign hyperplasia and dysplasia in HPV16 mice. Although immunocomplexes (ICs) accumulated in premalignant dermal stroma, the classic and alternative complement pathways, including the C3 complement, did not contribute to the neuroplastic progression. However, C5aR1^+^ cells and C5a deposition were consistently found in areas of high-grade dysplasia and squamous cell carcinomas (SCCs). Intradermal transplantation of HPV16 SCC-derived cell lines into C5aR1^+/−^ versus C5aR1^−/−^ syngeneic hosts showed significantly impeded tumor growth in C5aR1^−/−^ recipients. Diminished levels of VEGF and CD31^+^ vasculature density were observed in C5aR1^−/−^ mice with end-stage SCCs. Additionally, the premalignant skin of HPV16/C5aR^−/−^ mice displayed a weakened leukocyte infiltration. Lastly, HPV16/uPA^−/−^ was found to contain reduced deposition of C5a in dermal stroma and skin lysates. Therefore, the generation of C5a in vivo appears to be linked to a uPA-dependent mechanism. To verify this, the study implanted PDSC5 cells orthotopically in both uPA-wild type and uPA^−/−^ mice in which they observed that uPA-deficient mice experienced slower tumor progression. The combination of PMX-53, a C5aR1 antagonist, with paclitaxel chemotherapy synergistically slowed SCC growth. Depletion of CD8^+^ T cells, but not CD4^+^ T cells, demonstrated that CD8^+^ cells are required for the efficacy of PMX-53/PTX combination therapy [28].

Ding et al. recently developed a colitis-associated cancer (CAC) model by combining azoxymethane (AOM) and dextran sulfate sodium (DSS) in C57BL/6 mice deficient in C3, C5, C5ar1, C5ar2, or wild-type. The study showed that C5aR1 signaling, independent of C3 activation, plays a crucial role in CAC tumorigenesis by modulating immune responses. The study showed that deficiency in C5 and C5aR1 significantly prevented tumorigenesis. Moreover, PMX205, a C5aR1 antagonist, blocked AOM-DSS-induced colorectal cancer (CRC) tumorigenesis. Notably, complement activation was evident at inflamed colorectal sites, marked by the presence of C3d and C5a cleavage products. In wild-type mice, AOM/DSS treatment significantly elevated the proportion of myeloid-derived suppressor cells (MDSCs; CD11b^+^Gr-1^+^), which was reduced in C5- or C5aR1-deficient mice, but not in C3-deficient mice. In contrast, tumor tissues from C5- and C5aR1-deficient mice had increased infiltration of CD8^+^ T cells compared to wild-type and C3-deficient mice, suggesting that MDSCs may contribute to CD8^+^ T cell suppression and create an immunosuppressive tumor microenvironment. Bone marrow transplantation experiments demonstrated that C5aR1 expression in immune cells is sufficient to initiate CRC. Overall, the study suggests that C5aR1 is a key regulator of immune suppressive responses and a promising target for CRC prevention [27].

Luan et al. showed that tumor-associated macrophages (TAMs) driven by intratumoral C5a/C5aR signaling exhibit a pro-tumorigenic phenotype. This signaling pathway promotes tumor progression by suppressing CD8^+^ cytotoxic T lymphocyte infiltration through inhibition of CXCL9 production in TAMs, a chemokine crucial for recruiting cytotoxic T cells. In the study involving ovarian cancer cells inoculated into control and C5aR^−/−^ mice, macrophage proliferation was monitored. The results showed that C5aR did not significantly affect macrophage proliferation, but macrophages from the C5aR^−/−^ group exhibited enhanced cytotoxicity against tumor cells. Compared to wild-type mice, C5aR^−/−^ mice had increased M1 macrophages and decreased M2 macrophages in the tumor microenvironment (TME), with no significant changes in blood and spleen macrophage populations. These findings suggest that C5a/C5aR signaling contributes to M2 macrophage polarization in the TME. Notably, high C5aR levels were identified in ovarian cancer and in TAMs, which polarized them toward immunosuppressive phenotypes. C5aR deficiency or inhibition restored TAMs to an anti-tumor function by enhancing CD8^+^ cytotoxic T cell responses, dependent on CXCL9 secretion from TAMs. Furthermore, C5aR inhibition synergized with PD-1 blockade immunotherapy. The study further demonstrated that high C5aR expression significantly correlated with poor survival in various types of cancer. This study elucidates the immunosuppressive effects of the C5a/C5aR axis on TAMs, highlighting the potential of targeting C5aR for clinical applications [60].

Li et al. demonstrated that exosome-transmitted long noncoding RNA TMZ-associated lncRNA in glioblastoma recurrence (lnc-TALC) remodels the glioblastoma microenvironment and reduces tumor sensitivity to temozolomide (TMZ) chemotherapy. The study showed that Inc-TALC is packaged into exosomes and delivered to tumor-associated macrophages (TAMs), driving M2 polarization of microglia in a manner correlated with C5/C5a secretion. This process enables DNA damage repair caused by temozolomide (TMZ) treatment, resulting in chemotherapy resistance. Targeting C5aR with immunotherapy overcomes this resistance, enhancing TMZ efficacy by disrupting the Inc-TALC-mediated signaling pathway. The study showed that microglia exposed to exosomes from GBM cells overexpressing lnc-TALC had increased C5 expression at the transcriptional level, which was regulated by lnc-TALC binding to ENO1 and activating p38. Inhibition of p38 activity by SB203580 decreased C5 expression, highlighting the potential therapeutic target for overcoming TMZ resistance [61].

Gao et al. explored the impact of dysregulated circular RNAs (circRNAs) on anti-PD1 resistance in non-small-cell lung cancer (NSCLC). They discovered that overexpressing circASCC3 enhanced the malignant phenotype of NSCLC cells, creating an immunosuppressive microenvironment characterized by CD8^+^ T cell exhaustion and M2 macrophage polarization. Further study revealed that circASCC3 sponged miR-432–5p to increase complement C5a levels, which enhanced the progression and dysfunctional immune status of NSCLC. Combining PMX-53, a C5aR1 inhibitor, with anti-PD1 antibody suppressed NSCLC growth driven by circASCC3 and enhanced the efficacy of anti-PD1 monotherapy. The C5a/C5aR axis promotes an immunosuppressive environment through M2 macrophage polarization and increased PD-L1 expression on CD8^+^ T cells. The combination therapy prevented CD8^+^ T cell exhaustion, boosting anti-PD1 effectiveness. This synergistic approach shows promise for treating advanced NSCLC patients [62].

Zhang et al. investigated the role of C5aR1 in high-grade serous ovarian cancer (HGSC), focusing on prognosis, immune microenvironment, and immunotherapy response. They found that C5aR1 expression was elevated in the immunoevasive subtype of HGSC, characterized by increased pro-tumoral immune cell infiltration (Treg cells, M2-polarized macrophages, and neutrophils) and impaired CD8^+^ T cell function. High C5aR1 expression correlated with poor prognosis. Notably, inhibiting C5aR1 with PMX53 suppressed tumor growth, enhanced anti-tumor pathways, and improved PD-1 therapy efficacy [63].

**Figure 4:**
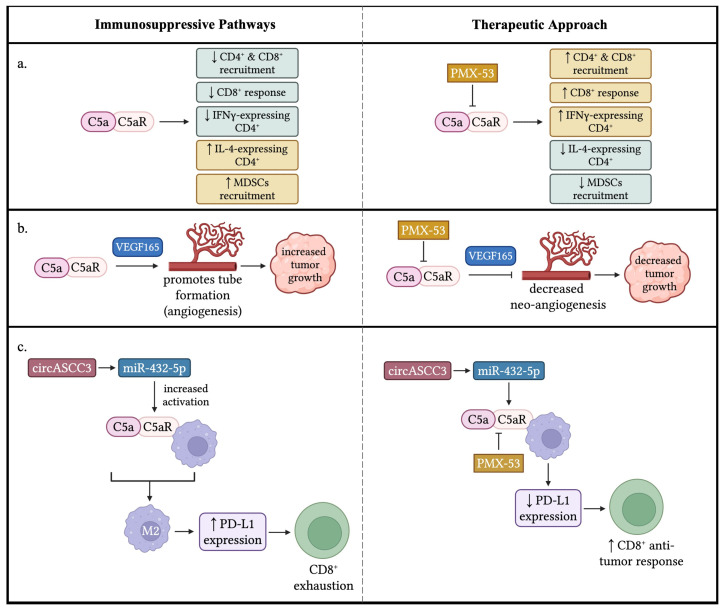
**Immune-suppressive effects of C5a/C5aR signaling in the TME.** (**a**) C5a/C5aR signaling results in numerous conditions that promote tumor growth: a decrease in CD4^+^ and CD8^+^ recruitment, a decrease in CD8^+^ response (i.e., increased exhaustion), a decrease in CD4^+^ cells that express IFN-*γ*, an increase in CD4^+^ expressing IL-4, and an increase in the recruitment of MDSCs. Thus, a potential treatment would result in a change of these factors to reduce tumor growth by inhibiting C5a/C5aR signaling. (**b**) C5a/C5aR signaling plays a role in neo-angiogenesis. C5a promotes the formation of new vessels within the TME to support tumor growth through a VEGF165-dependent manner. A therapeutic approach could target C5a/C5aR signaling to impede new tube formation within the TME, consequently decreasing tumor growth. (**c**) circASCC3 promotes miR-432-5p to increase the levels of C5a, resulting in an increased activation of C5a/C5aR signaling with macrophages. The anaphylatoxin signaling induces macrophages to adopt an M2 phenotype, leading to an increased expression of PD-L1 that results in the exhaustion of CD8^+^ T cells. A potential therapeutic approach, such as a PMX-53 blockade C5a/C5aR, would diminish the numbers of macrophages that adopt an M2 phenotype and decrease the expression of PD-L1, producing a CD8^+^ anti-tumor response. ↓: decrease; ↑: increase. The figure was created with BioRender.com.

Ortiz-Espinosa et al. recently demonstrated that C5a enhances the ability of polymorphonuclear myeloid-derived suppressor cells (PMN-MDSCs) to promote tumor growth and metastasis by inducing neutrophil extracellular trap (NET) formation. This process is dependent on cancer cell production of high-mobility group box 1 (HMGB1) and C5a-induced expression of HMGB1 receptors TLR4 and RAGE on PMN-MDSCs. Notably, inhibiting C5a, C5a receptor-1 (C5aR1), or NETosis in a lung metastasis model reduced circulating tumor cells and metastatic burden. Furthermore, elevated levels of myeloperoxidase (MPO)-DNA complexes, markers of NETosis, were observed in lung cancer patients and correlated with C5a levels, suggesting that C5a promotes NET formation and potentially facilitates cancer dissemination and metastasis [64]. The immunologic regulation of the C5a/C5aR signaling pathway in TME is summarized in Table 4 and Figure 4.

## 5. Anaphylatoxin Signaling Pathways Inhibit Tumor Progression by Modulating Immune Cell Functions Within the TME

Although extensive research supports the role of anaphylatoxin signaling pathways in driving cancer progression, some studies present conflicting data, as illustrated in the following examples. Gunn et al. found that C5a plays a complex role in tumor progression, with differing effects depending on the context. In an immunocompromised mouse model, C5a-expressing lymphoma tumors grew slower and increased infiltration of NK cells and macrophages, which are important for tumor immunity. The tumor microenvironment also showed decreased production of pro-tumorigenic factors like VEGF, arginase, and TNF-α. However, in an immunocompetent mouse model, C5a-expressing lymphoma tumors grew faster and increased frequencies of myeloid-derived suppressor cells (MDSCs) and regulatory T cells (Tregs), while CD4^+^ and CD8^+^ T cell frequencies were reduced. The study indicates that C5a’s effects on tumor growth depend on its concentration in the tumor microenvironment. For instance, a high C5a concentration provokes tumor growth through exacerbated inflammation by stimulating activation of infiltrating cells. Furthermore, a high C5a concentration enhances a pro-tumoral microenvironment, as it correlates with induced T_reg_ differentiation and suppressed T_h_1 cell differentiation, decreasing anti-tumoral activity [12].

Kim et al. found that expressing C5a in the EMT6 murine mammary tumor model, which is poorly immunogenic, resulted in slower tumor growth and complete regression in one-third of mice with C5a-expressing tumors. Notably, mice that experienced tumor regression developed complete immunity to subsequent challenges with unmodified tumor cells. The study suggests that C5a plays a positive role in triggering an anti-tumor response, characterized by increased tumor infiltrates, particularly activated macrophages and granulocytes. These immune cells can directly or indirectly kill tumor cells through mechanisms like phagocytosis, cytokine secretion, or reactive oxygen production. The involvement of acquired immunity is also evident, as mice that rejected primary tumors were immune to subsequent challenges, potentially implicating dendritic cells in post-rejection immunity. Intriguingly, this anti-tumor effect was observed only in cells expressing relatively low levels of C5a, not in those with high C5a expression [11].

An analysis by Akhir et al. utilized primary murine mammary carcinoma models to demonstrate diminished tumorigenesis due to a dual C3aR/C5aR1 agonist (YSFKPMPLaR). The anti-tumoral effects of the agonist are regulated by the modulation of immune cell functions, as evidenced by the low mRNA expression of C3aR and C5aR1 detected in EMT6 and 4T1 mammary carcinoma cell lines by qPCR analysis. Therefore, the agonist treatment resulted in a significant elevation of T lymphocytes, demonstrating an immunoregulatory role of C3aR and C5aR1. These findings show that complement activation peptides can have diverse impacts on anti-tumor responses, influenced by factors such as cancer type, host immune response, and the level of endogenous complement activation within the tumor microenvironment [13].

Bandini et al. examined the contribution of the C3 complement in autochthonous mammary carcinoma progression. In a knockout mice model, neuT-C3^−/−^ (C3^−/−^ male mice crossing with female mice expressing Her2/neu oncogene), was utilized to demonstrate an accelerated tumor onset from upregulated Her2/neu oncogene expression. Additionally, the study recorded a marked elevation of Tregs within the tumor microenvironment. NeuT-C3^−/−^ malignant cells were transplanted into immunocompetent hosts, resulting in lesions with delayed kinetics and diminished carcinogenesis [14].

All of these studies displayed conflicting results, revealing that anaphylatoxins C3a and C5a might promote immune functions against tumor progression in TME [11,12,13,14]. The impact of the complement system on tumor progression likely depends on various factors, including genetic background, oncogene characteristics, immunological tolerance to transgene products, tumor type, and penetrance, leading to diverse outcomes. Elucidating the molecular specifics of this key immunoregulatory component in the tumor microenvironment could enable harnessing complements as a major contributor to immunosurveillance and cancer control in clinical settings.

## 6. Perspectives and Future Directions

Early studies presented that C3a and C5a anaphylatoxins engage their respective receptors on dendritic cells (DCs) and CD4^+^ T cells, providing costimulatory and survival signals essential for effector T cell responses under normal physiological conditions [65,66,67]. These receptors are also required for T_h_1 and T_h_17 cell differentiation, mediating IL-12 production by DCs and IL-12 receptor expression by CD4^+^ T cells, as well as IL-6 and IL-23 production [68,69]. Collectively, anaphylatoxins C3a and C5a are vital for DC and T cell function in normal physiological conditions. However, in cancer-bearing mice models, C3a and C5a surprisingly shift to suppressing effects on immune cells function and promote tumor progression. The underlying mechanism for this remains unclear. Although most recent studies displayed a paradigm shift on the role of C3a and C5a in cancer progression, some studies demonstrated that they still could inhibit cancer progression by boosting immune functions, similar to their roles under normal physiological conditions [11,12,13,14]. For example, Kim et al. found that expressing C5a in poorly immunogenic EMT6 murine mammary tumors caused complete regression in one-third of mice that were xenografted with EMT6 tumor cells and triggered anti-tumor immune responses. Notably, this anti-tumor effect was observed only at lower C5a expression levels, indicating a dual role for C5a depending on its expression level in the tumor microenvironment (TME). The dual role of C5a has been demonstrated in various disease models, such as infection, autoimmune diseases, organ transplantation, and allergy [70,71,72,73]. Another study in a cancer model has also demonstrated complex roles for C5a in tumor progression, with varying effects on immune cell infiltration and tumor growth [12]. Additionally, C3aR and C5aR agonists have been shown to slow mammary tumor development and progression through regulations of immune cell responses [13]. C3 deficiency has been demonstrated to be associated with accelerated tumor onset and increased Treg cell frequency in neuT mice [14]. In summary, the anaphylatoxins’ impact on tumor progression is likely influenced by multiple factors, including genetic background, oncogene characteristics, immunological tolerance, tumor type, and penetrance, resulting in varied outcomes. Elucidating the molecular role of anaphylatoxins in the tumor microenvironment may enable researchers to tap into their therapeutic potential for enhancing immunosurveillance and cancer control.

In tumor-bearing mice model, extensive studies demonstrated that anaphylatoxins C3a and C5a inhibit immune cell functions in TME by recruiting and inhibiting immune cell functions. The crosstalk between anaphylatoxins with their cognate receptors on immune cells in TME appears complex, since anaphylatoxins’ receptors are expressed in various immune cells. C3aR and C5aR are expressed on various myeloid-derived innate immune cells, including basophils, dendritic cells, eosinophils, macrophages, mast cells, and monocytes [74,75,76,77,78,79,80,81,82,83]. Inflammatory mediators and complement factors play a key role in regulating the expression of those receptors on myeloid cell [84,85,86]. Anaphylatoxin receptors are also found on T cells [65,66,87,88,89], where they modulate subset differentiation, including T_h_1, T_h_2, T_h_17, and Treg cells, although study findings have been inconsistent [66,68,90,91,92].

Current research is focused on evaluating the effectiveness of combining anaphylatoxin-targeted therapies with standard cancer treatments, such as chemotherapy, radiotherapy, and immune checkpoint inhibitors. Notably, anaphylatoxins like C3a and C5a can exert immunosuppressive effects within the tumor microenvironment (TME), suggesting that inhibiting their signaling pathways may improve immune cell function and enhance anti-tumor responses. Research has shown promising outcomes with the combination of PMX-53, a C5aR1 antagonist, and paclitaxel chemotherapy, resulting in slowed growth of squamous cell carcinoma. This approach highlights the potential therapeutic benefits of targeting the C5a pathway in conjunction with standard chemotherapy [28]. Further research is needed to explore the optimal dosing and scheduling of PMX-53 in combination with chemotherapy and to investigate its potential applications in other cancer types. A recent study by O’Brien found that radioresistant rectal cancer cells exhibit increased expression of C3 and C5, as well as their activation products, anaphylatoxins C3a and C5a. Inhibiting C3 increases DNA damage and shifts cells towards a radiosensitive phenotype, making them more susceptible to radiation treatment. Furthermore, high levels of C3a and C5b-9 in pre-treatment sera from rectal cancer patients are associated with poor response to neoadjuvant chemoradiotherapy (neo-CRT) and poor prognosis, indicating that measuring levels of C3a and C5b-9 in patient sera may help predict treatment response and identify patients who could benefit from the combination therapies [93]. Similar results were observed in locally advanced esophageal adenocarcinoma (EAC) patients treated with neoadjuvant chemotherapy. The study revealed that non-responders (NRs) exhibited high levels of C3a and C5a within their plasma prior to anticancer therapy compared to complete responders (CRs). Therefore, the anaphylatoxins may play a role in recruiting immunosuppressive cells to the tumor microenvironment [94,95]. Recent studies have further explored the therapeutic potential of combining anaphylatoxin inhibition with other treatments, like checkpoint inhibitors. Dual blockades of C3aR and C5aR using SB 290157 and PMX205 suppressed melanoma tumor growth, with additional benefits observed when they are paired with anti-PD-1 blockade [7,56]. Targeting C3aR alongside anti-PD-1 treatment also demonstrated potential for reducing primary tumor growth in certain sarcomas [19]. Ajona et al. found that concurrent blockade of C5a/C5aR1 and PD-1 signaling synergistically reduced tumor growth and metastasis, leading to prolonged survival in preclinical animal models. The effect was accompanied by a shift in the tumor immune landscape, with a negative association between CD8^+^ T cells and myeloid-derived suppressor cells [15]. Furthermore, Yuan et al. showed that radiotherapy increases C5aR expression and CD8^+^ T cell infiltration, and that blocking C5aR with W-54011 can improve radiotherapy sensitivity in non-small-cell lung cancer [16]. These findings collectively suggest that inhibiting anaphylatoxins C3a and C5a can synergize with various cancer treatments, enhancing therapeutic efficacy and offering new avenues for cancer therapy.

## 7. Conclusions

In most cases, the complement system’s components, C3a and C5a, play a pivotal role in promoting cancer progression and metastasis by enhancing tumor growth and suppressing anti-tumor immunity. Elucidating the mechanisms underlying their involvement in cancer development offers a promising avenue for therapeutic innovation. Specifically, targeting C3a and C5a with inhibitors may provide a novel strategy to augment cancer treatment efficacy. Further research into the complex interplay between the complement system and cancer is warranted to fully exploit the therapeutic potential of these signaling pathways.

## Figures and Tables

**Table 1:** **Generation of anaphylatoxins C3a and C5a through canonical and noncanonical mechanisms in the tumor microenvironment (TME)**.

Cancer Model	Complement Activation in the TME	References
Cervical cancer	Classical or lectin complement pathway activation involves C5a production in TME. C1q deposition patterns suggested the classical pathway’s involvement. Tumor growth was reduced in C3 and C4 deficient mice, but not in factor B deficient mice.	Markiewski et al. [8]
Sarcoma	The lection complement pathway is activated by sarcomagenesis. Genetic deficiencies in C3, C4, and MBL1/2 delayed tumor appearance, whereas C1q and factor B deficiencies had little to no impact.	Magrini et al. [19]
Glioblastoma (GBM)	Bb-dependent complement activation of the alternative complement pathway. Abundant C3a staining and robust staining for Bb neoepitope were found in GBM.	Ah-Pine et al. [20]
Lung cancer	A non-canonical bypass routes by trypsin-like serine protease. SBTI and TLCK, inhibitors of trypsin-like serine proteases, significantly reduced the local production of C5a.	Corrales et al. [23]
Epithelial ovarian cancer (EOC)	The classical or lectin complement activation pathway is involved in the progression of EOC. Either C3 or C5aR deficiency resulted in profoundly impaired EOC growth and reduced tumor vascularization, suggesting C3-dependent C5a production during EOC progression.	Nunez-Cruz et al. [33]
Squamous Carcinoma	C5 was cleaved in C3-independent manner by plasmin activated by urokinase (uPA)-expressing macrophages promotes carcinogenesis, Highlighting non-canonical activation of C5 via uPA.	Medler et al. [28]
Colorectal Cancer	Intracellular C5 is cleaved by cathepsin D (CTSD) to produce C5a in lysosomes and endosomes of colonic cancer cells. Intracellular C5a/C5aR1-mediated β-catenin stabilization. C5a/C5aR1 signaling drives colorectal tumorigenesis; C5-deficient mice show reduced tumorigenesis, whereas C3 deficiency does not affect, suggesting cascade-independent C5 activation is involved.	Ding et al. [9] Ding et al. [27]

**Table 2:** **The direct effects of anaphylatoxin C3a and C5a on tumor progression**.

Cancer Type	Mechanism Summary Additional Notes	References
Breast Cancer	High C5aR expression in tumor tissue; C5aR knockout or antagonist reduces tumor growth by modulating MAPK/p38 and p21 signaling. Noting the limitation of animal model due to the complexity of immune system involved.	Chen et al. [34]
Multiple myeloma	The expression of C3a and C5a receptors on myeloma cells of MM patients was also significantly higher than that on plasma cells of normal donors. C3a and C5a increase the migration, invasion and adhesion of MM cell lines by activating the MEK/ERK pathway and increasing the nuclear transfer of Nrf2.	Xiong et al. [36]
Colorectal cancer	Complement C5a generated intracellularly interacts with C5aR1 to stabilize β-catenin via KCTD5/cullin3/Roc-1 complex, linked to poor prognosis. Targeting C5a/C5aR may improve immunotherapy outcomes, Linking C5a/C5aR1 to β-catenin pathway and colorectal cancer prognosis.	Ding et al. [9] O’Brien et al. [10]
Anaplastic Thyroid Carcinoma (ATC)	Elevated C5aR1 promotes proliferation, migration, invasion via TLR1/2 and MyD88 pathway; miR-335-5p negatively regulates C5aR1, indicating Potential therapeutic axis: miR-335-5p/C5aR1/TLR1/2.	Liu et al. [37]
Multiple epithelial cancers (breast cancer, cervical cancer, Prostate cancer, keratinocytes, hepatocellular carcinoma)	C5a/C5aR interaction drives cancer cell proliferation, invasion, and checkpoint inhibitor expression. C5aR expression found on cancerous epithelial cells but not on normal tissue, suggesting induction by malignant transformation, highlighting heterogeneity of receptor expression. Inflammation may stimulate C5aR expression.	Nitta et al. [41] Imamura et al. [42] Yoneda et al. [43] Imamura et al. [44] Zwirner et al. [39] Buchner et al. [40]

**Table 3:** **Immunologic regulation of the C3a/C3aR signaling pathway in TME**.

Cancer Type	Mechanism Summary	References
Glioblastoma	C3a and C3aR upregulated in TAMs; C3aR^+^ TAMs express M2 marker CD163 and VEGF; TGF-β1 upregulates VEGF, C3, and C3aR in TAMs promoting angiogenesis and immunosuppression. C3a recruits and polarizes macrophages to M2 phenotype; targeting C3aR in TAMs may reduce immunosuppression.	Ah-Pine et al. [20]
Melanoma, head and neck cancer	Tumor stromal S1 population expresses C3; C3a/C3aR signaling recruits C3aR^+^ macrophages; blocking C3a reduces macrophage infiltration and increases CD8^+^ T cells, slowing tumor growth. CAF-macrophage crosstalk via C3a/C3aR axis conserved in mice and human tumors; anti-C3a slows tumor growth.	Davidson et al. [54]
Sarcoma (3-MCA, MN/MCA1, FS6)	C3aR signaling is critical for complement-mediated sarcoma promotion and metastasis; C3, C4, MBL1/2 deficiencies delay tumor onset; C3aR deficiency reduces macrophage recruitment and increases M1 polarization and CD8^+^ T cells. C3aR expressed mainly on myeloid cells; C3 or C3aR deficiency enhances response to anti-PD-1 immunotherapy; combination therapy promising.	Magrini et al. [19]
Medulloblastoma	C3a activates astrocytes via p38 MAPK and TNF-α; promotes tumor growth; blocking C3aR or TNF-α inhibits tumor progression. High C3/C3a in tumor tissue; TNF-α receptor expression correlates with poor prognosis.	Gong et al. [38]
Breast cancer metastasis	C3a binding to C3aR activates PI3K/AKT signaling, leading to CAF activation. Blocking C3aR signaling genetically or pharmacologically inhibits lung metastasis of breast cancer.	Shu et al. [55]
Melanoma (B16 model)	C3-deficient mice show slower tumor growth; C3aR and C5aR suppress IL-10 production in CD8^+^ T cells; The blockade of C3aR and C5aR restores IL-10 and enhances anti-tumor immunity. Combined blockade of C3aR and C5aR with anti-PD-1 improves anti-tumor response; C3aR and C5aR act as immune checkpoint receptors on CD8^+^ TILs.	Wang et al. [7,56]
Melanoma (B16-F0 model)	C3aR deficiency or antagonism reduces tumor growth; increases neutrophils and CD4^+^ Th1, Th2, Th17 cells; decreases macrophages; neutrophils essential for anti-tumor effects. C3aR blockade slows growth of melanoma, colon, and mammary carcinoma models; potential broad therapeutic target.	Nabizadeh et al. [53]
Colorectal carcinoma, Melanoma, breast cancer, lung cancer, lymphoma	Tumor cell–derived C3 was activated intracellularly, which results in generation of C3a. C3a modulated tumor-associated macrophages via C3a/C3aR-PI3Kg signaling, thereby repressing anti-tumor immunity. Deletion of C3 in tumor cells that had high C3 expression enhanced efficacy of anti–PD-L1 treatment.	Zha et al. [57]
Cutaneous squamous cell carcinoma (cSCC)	The absence of C3 conferred protection against skin cancer development in the DMBA-TPA model of cSCC. The findings suggest that C3 activation products, such as iC3b, C3b, and C3d, rather than anaphylatoxins C3a and C5a, can promote tumor growth and epithelial hyperplasia by interacting with the receptor CR3 on infiltrating myeloid cells.	Jackson et al. [58]

**Table 4:** **Immunologic regulation of the C5a signaling pathway in TME**.

Cancer Type	Mechanism Summary	References
Cervical Cancer (TC-1 model)	C5a in TME suppresses CD8^+^ T cell anti-tumor response; promotes MDSC recruitment and their immunosuppressive functions; blocking C5aR enhances CD8^+^ T cells and reduces tumor growth. Classical/lectin pathway implicated; C5a acts on host cells; MDSCs produce ROS and RNS.	Markiewski et al. [8]
Non-Small-Cell Lung Cancer (NSCLC)	Lung cancer cells produce higher C5a; C5a promotes angiogenesis and immunosuppressive TME; blocking C5aR reduces tumor growth and MDSC levels, lowers immunosuppressive gene expression. C5a does not directly affect cancer cell proliferation; C5aR blockade could enhance cancer therapies.	Corrales et al. [23]
Epithelial Ovarian Cancer (EOC)	C3 deficiency impairs tumor growth; increased CD8^+^ T cells and decreased FoxP3^+^ CD4^+^ T cells; C5a promotes VEGF165-dependent tube formation; C5aR deficiency impairs tumor growth and angiogenesis. C3 and C5aR roles in angiogenesis and immune regulation.	Nunez-Cruz et al. [33]
Ovarian Cancer	C5a shows context-dependent effects and slows tumor growth in immunocompromised mice with increased NK/macrophage infiltration; promotes tumor growth in immunocompetent mice via increased MDSCs and Tregs. Effects depend on C5a concentration; high levels promote Treg induction and suppress Th1.	Gunn et al. [12]
Mammary Carcinoma (EMT6 model)	C5a expression slows tumor growth and induces tumor regression; triggers anti-tumor immunity via macrophages and granulocytes; low C5a expression beneficial. Induced long-term immunity to rechallenge, implicating acquired immunity.	Kim et al. [11]
Mammary Carcinoma (EMT6 and 4T1 models)	Dual C3aR/C5aR1 agonist slows tumor progression; increases T lymphocytes suggesting immunoregulatory roles. Low receptor mRNA in cell lines indicates immune-mediated effects.	Akhir et al. [13]
Mammary Carcinoma (neuT model)	C3 deficiency accelerates tumor onset with increased Tregs; transplanted tumor cells from C3^−/−^ mice grow slower in immunocompetent hosts. Complement impact depends on genetic background, oncogene, immune tolerance, tumor type.	Bandini et al. [14]
Breast Cancer	C5aR promotes metastasis by suppressing T cell responses; recruits MDSCs and Tregs, increases immunosuppressive cytokines; blockade enhances Th1/Tc1 responses and reduces metastasis. MDSCs and complement deposition found in patient lymph nodes.	Vadrevu et al. [59]
Colitis-Associated Cancer (CAC)	C5aR1 signaling crucial for tumorigenesis, independent of C3; deficiency or antagonist prevents tumor development; modulates MDSC and CD8^+^ T cell infiltration. Bone marrow C5aR1 expression sufficient to initiate CRC tumorigenesis.	Ding et al. [27]
Ovarian Cancer	C5a/C5aR signaling in TAMs promotes M2 polarization, suppresses CXCL9, impairs CD8^+^ T cell infiltration; C5aR deficiency shifts TAMs to M1 phenotype; synergizes with PD-1 blockade. High C5aR correlates with poor survival; therapeutic potential.	Luan et al. [60]
Glioblastoma	Exosome-transmitted lnc-TALC promotes M2 microglia polarization via increased C5/C5a secretion, causing temozolomide resistance; C5aR antagonist improves therapy. lnc-TALC regulates C5 via ENO1 and p38; p38 inhibition reduces C5.	Li et al. [61]
Non-Small-Cell Lung Cancer (NSCLC)	circASCC3 overexpression increases C5a, induces M2 macrophage polarization, CD8^+^ T cell exhaustion, and PD-L1 expression; C5aR1 inhibitor PMX-53 + anti-PD1 improves therapy. C5a/C5aR axis drives immunosuppressive microenvironment and resistance.	Gao et al. [62]
High-Grade Serous Ovarian Cancer (HGSC)	High C5aR1 expression correlates with poor prognosis, increases pro-tumor immune cells, impairs CD8^+^ T function; C5aR antagonist PMX53 reduces tumor growth and synergizes with anti-PD-1. C5aR1 is an independent prognostic factor and therapeutic target.	Zhang et al. [63]

## Data Availability

No new data were created or analyzed in this study. Data sharing is not applicable to this article.
